# GEVALT: An integrated software tool for genotype analysis

**DOI:** 10.1186/1471-2105-8-36

**Published:** 2007-02-01

**Authors:** Ofir Davidovich, Gad Kimmel, Ron Shamir

**Affiliations:** 1 School of Computer Science, Tel-Aviv University, Tel-Aviv, Israel

## Abstract

**Background:**

Genotype information generated by individual and international efforts carries the promise of revolutionizing disease studies and the association of phenotypes with alleles and haplotypes. Given the enormous amounts of public genotype data, tools for analyzing, interpreting and visualizing these data sets are of critical importance to researchers. In past works we have developed algorithms for genotypes phasing and tag SNP selection, which were shown to be quick and accurate. Both algorithms were available until now only as batch executables.

**Results:**

Here we present GEVALT (GEnotype Visualization and ALgorithmic Tool), a software package designed to simplify and expedite the process of genotype analysis, by providing a common interface to several tasks relating to such analysis. GEVALT combines the strong visual abilities of Haploview with our quick and powerful algorithms for genotypes phasing (GERBIL), tag SNP selection (STAMPA) and permutation testing for evaluating significance of association. All of the above are provided in a visually appealing and interactive interface.

**Conclusion:**

GEVALT is an integrated viewer that uses state of the art phasing and tag SNP selection algorithms. By streamlining the application of GERBIL and STAMPA together with strong visualization for assessment of the results, GEVALT makes the algorithms accessible to the broad community of researchers in genetics.

## Background

Genotype information generated by individual and international efforts carries the promise of revolutionizing disease studies and the association of phenotypes with alleles and haplotypes. Given the enormous amounts of public genotype data, tools for analyzing, interpreting and visualizing these data sets are of critical importance to researchers.

In past works we have developed the following analysis algorithms:

**1. GERBIL **[1,2] – an algorithm for simultaneously phasing genotypes into haplotypes and block partitioning. The algorithm is based on a stochastic model for recombination-poor regions ("blocks"), in which haplotypes are generated from a small number of core haplotypes, allowing for mutations, rare recombinations and errors. The genotype phasing and block partitioning is solved by an expectation-maximization algorithm. Gerbil accepts genotype data as input and outputs the phased genotypes for each individual, the block structure of the entire population and the common haplotypes in each block. As part of the algorithm, Gerbil also accurately completes missing data according to the common haplotypes found. Gerbil was shown to be quick and accurate even for many hundreds of individuals [1].

**2. STAMPA **[3] – an algorithm for tag SNP selection. The algorithm finds a set of tag SNPs with maximal prediction accuracy. The prediction accuracy of a set of tag SNPs is the expected accuracy of predicting untyped SNPs, given the tag SNPs. Dynamic programming is used in order to efficiently find the set of tag SNPs. Halperin et. al tested Stampa on many different genotype datasets from different sources, and showed that it finds tag SNPs with considerably better prediction ability than two other state-of-the-art tag SNP selection algorithms [3].

Both GERBIL and STAMPA were available until now only as batch executables. In this work we introduce GEVALT (GEnotype Visualization and ALgorithmic Tool). GEVALT (Version 1.1) is an integrated software providing easy access to the GERBIL and STAMPA algorithms as well as to some other tools for genotype analysis. GEVALT is based on Haploview version 3.2 [4] and it maintains the user-friendly interface and strong visualization capabilities of Haploview, as well as its other functionalities, including computation of marker quality statistics and LD information.

## Implementation

GEVALT is implemented in JAVA based on the open source code of Haploview version 3.2. The analysis algorithms (GERBIL, STAMPA and permutation testing) are implemented in C++. Both Linux and Windows versions of GEVALT are available for download, as well as the JAVA source code.

## Results and Discussion

GEVALT accepts input in a variety of formats. Genotype data can be loaded as unphased genotypes in the standard linkage format, or as either partially or fully phased chromosomes. Genotype data dumps from the HapMap website [5] can also be loaded. When using the standard linkage format, the user can specify family structure as well as disease status. The user can also specify marker information, including name and location. Upon loading a dataset, GEVALT first phases the genotypes in the following manner: For data consisting of unrelated individuals, GEVALT uses Gerbil to phase the genotypes. For data consisting of two-generation pedigrees, GEVALT first creates a set of trios, one per family, where each trio contains the child with least missing data. In each trio phasing is done, if possible, according to Mendelian rules. Then only the children's genotypes are phased using Gerbil and each parent's haplotypes are deduced from its child's haplotypes. Gerbil is then run again on the set of the parents' genotypes only to complete the missing data. The haplotypes of the children that were not included in the trios are deduced from their parents' haplotypes.

**Figure 1:**
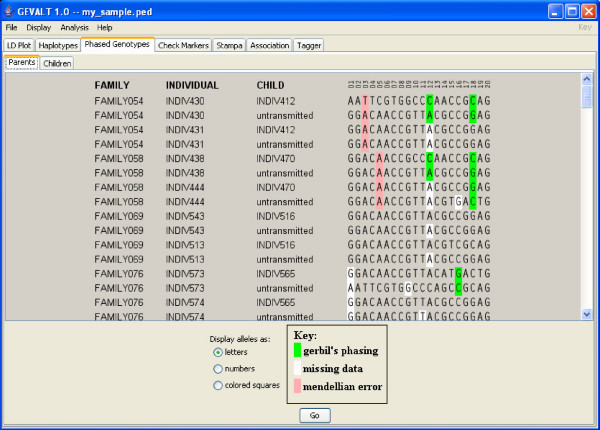
**The Phased Genotypes display**. The phased genotypes display is divided into two tabs – parents and children. In this image the parents tab is displayed. For each parent, its two chromosomes are displayed, with an indication which of them was transmitted to the child that was included in the trio. Different colors are used to indicate alleles phased by GERBIL, missing data that were completed by GERBIL, and Mendelian errors.

**Figure 2:**
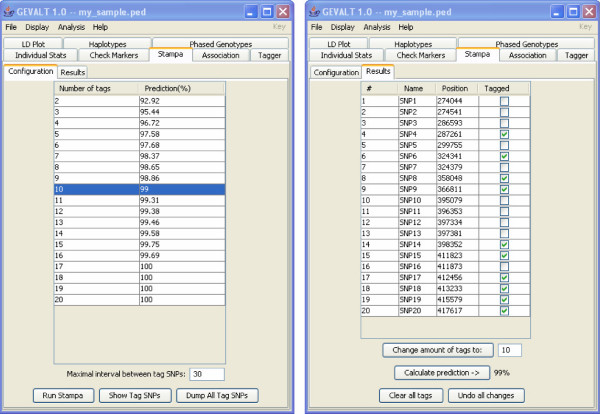
**The Stampa display**. Left – The Stampa configuration menu. After running Stampa, a table is displayed, showing for every number of tag SNPs, the prediction accuracy of the best set of tag SNPs of that size. The user can choose the number of tags to display according to the required prediction accuracy. Right – The Stampa results menu. The selected tag SNPs are marked. The user can add or remove tag SNPs manually and recalculate the prediction accuracy of the new set.

**Figure 3:**
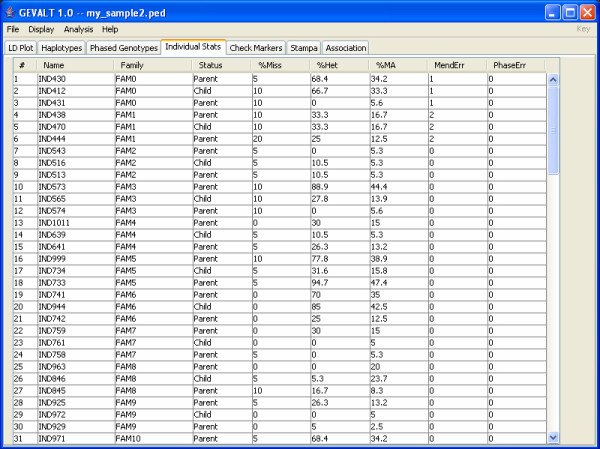
**The Individual Stats display**. This table summarizes statistics for each individual. These include the percentage of missing genotypes, the percentage of heterozygous markers, the percentage of minor alleles, and a tally of Mendelian inheritance errors and of phasing errors.

After phasing is completed, GEVALT generates several displays and option menus including the following:

• **Phased genotypes**: The phased genotypes of each individual are displayed. Different colors are used to indicate alleles phased by GERBIL, missing data and Mendelian errors (Figure 1). For data consisting of pedigrees, the phased genotypes are divided into two groups, parents and children, and each group is displayed separately.

• **Stampa**: Select tag SNPs using the Stampa algorithm (Figure 2). The user can specify the desired number of tag SNPs, and the algorithm finds an optimal set of tag SNPs and reports its prediction accuracy. The user can add or remove tag SNPs from the set manually and GEVALT recalculates the prediction accuracy of the new set.

• **Individual Stats**: Summary statistics for each individual are displayed (Figure 3). These include the percentage of missing genotypes, the percentage of heterozygous markers, the percentage of minor alleles and a tally of Mendelian inheritance errors.

**Figure 4:**
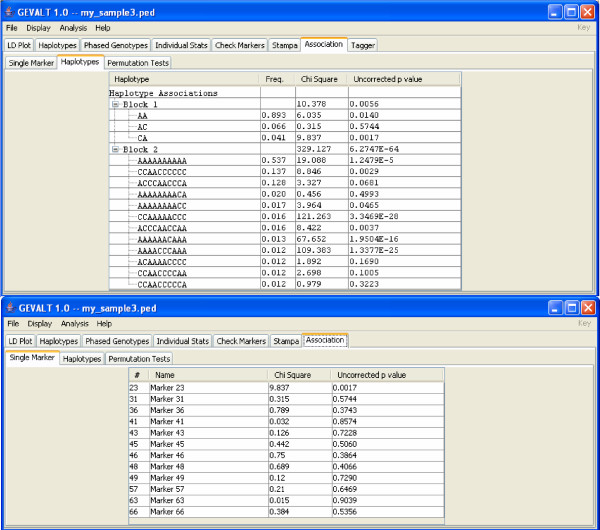
**The Haplotypes and Single Markers Associations displays**. The Association tab contains three displays: Single Marker, Haplotypes, Permutation Tests. Top – The Haplotypes Association tab. For each block and each haplotype in a block a chi-square score is calculated (TDT test for pedigrees, case/control test for unrelated individuals) and a p-value is derived. Only haplotypes above a certain frequency threshold are considered and displayed (the threshold is set by the user in the Haplotypes tab). Bottom – The Single Marker tab for the same set of markers. In this example more significant associations are detected when testing for haplotype associations than when testing individual SNPs.

**Figure 5:**
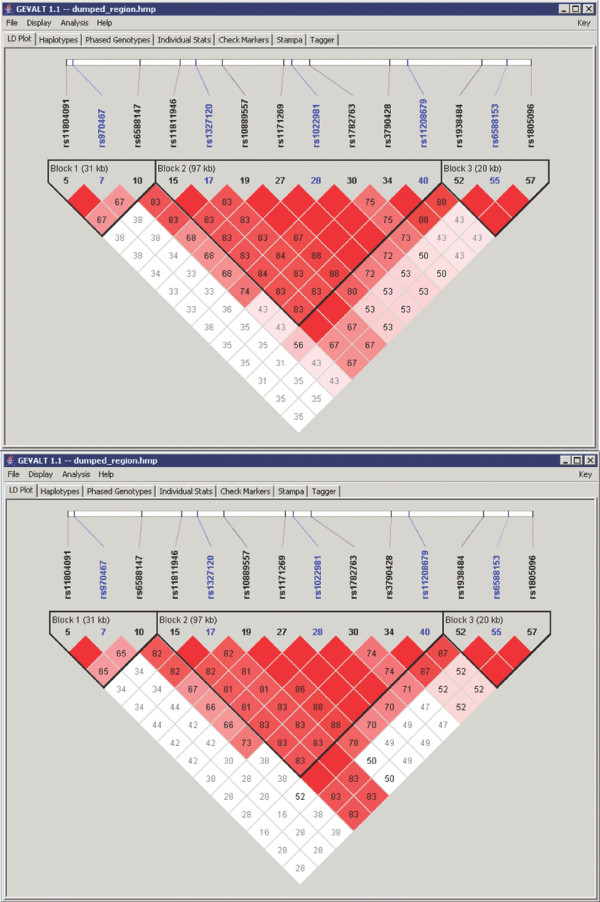
**LD plots comparison**. Top – An LD plot calculated using the phased genotypes (default). Bottom – An LD plot calculated using the unphased genotypes. Different LD scores are observed in many SNP pairs. These differences result from unambiguous phasing of double heterozygotes and from completed missing data. Markers that were chosen as tag SNPs in the Stampa tab are highlighted in blue.

All of Haploview's displays and option menus are available. See [4] for a full description of these features. In addition, the following changes and extensions are introduced:

• **Association**: GEVALT contains a faster implementation of the permutation test in C++ instead of JAVA. The new implementation runs about 20 times faster than the JAVA implementation in Haploview. In the haplotype associations tab, a p-value is calculated for each block and for each haplotype (Figure 4).

• **LD Plot**: The LD between each pair of SNPs can be calculated using either the phased or the unphased genotypes (Figure 5). The block structure displayed by default is that found by GERBIL. The user can still employ any of Haploview's block identification methods or select blocks manually. Markers that were chosen as tag SNPs in the Stampa tab are highlighted in this display.

• **Haplotypes**: The common haplotypes in each block are computed based on the Gerbil solution.

• **Check markers**: Phasing by Gerbil is done only for the set of picked markers. Whenever the set of picked markers changes, GEVALT recalculates the phasing, and all displays are updated accordingly.

Each of these displays and option menus is shown on a separate tab, allowing the user to move from one to the next. Interactive modifications made by the user in any panel are reflected in all the others. The information on each panel can also be exported to a PNG image file or to a text file. Additionally, the program has a command-line mode, which allows the user to run all the analyses without opening the GUI on one or more files at once.

The running time of GEVALT is dominated primarily by that of Gerbil and Stampa (see the references for detailed reports on the running times of these programs). All other operations, such as parameter adjustments and display changes, are done with no noticeable delay even for data sets with hundreds of markers and hundreds of individuals. Gerbil can currently handle up to 300 markers, while Stampa can handle thousands of markers. Both algorithms can handle thousands of individuals.

To the best of our knowledge, only two extant programs offer both algorithmic and visualization tools for genotype analysis: Haploview [4] and HapScope [6]. As described above, GEVALT maintains the popular, user-friendly interface of Haploview, but replaces its standard EM algorithm for phasing with the Gerbil algorithm. This allows a more accurate estimation of the phased haplotypes and a visualization of each individual's inferred haplotypes (and not just common haplotypes as in Haploview). Besides the Tagger algorithm for tag SNPs selection implemented in Haploview (and also in GEVALT), GEVALT also offers STAMPA, which is not only very efficient, but also allows the user to choose the amount of tag SNPs. Other advantages and improvements over Haploview are listed above. A current limitation of Gerbil is allowing at most 300 markers. We intend to remove this limitation in the future (see below). The HapScope software includes analysis programs and a visualization tool. Most of the analyses are done separately using the command line and the results are then loaded into the visualization tool. In contrast, in GEVALT all the analyses are done within the graphical user interface, which makes it more user friendly and easy to use. HapScope uses PHASE [7] or SNPHAP [8] as its phasing algorithm. PHASE was shown to be slightly more accurate than Gerbil but much slower [1]. In contrast to HapScope, GEVALT facilitates association tests and can handle family structures. On the other hand, only HapScope includes modules for reference sequence annotation, SNP mapping and SNP classification.

We intend to continue the development of GEVALT. In particular, we shall extend Gerbil to handle more SNPs, and improve Stampa so that it incorporates into its solution predefined tag SNPs. We also intend to incorporate a new algorithm for evaluating the significance of disease association, which is dramatically faster than the standard permutation test [9].

## Conclusion

GEVALT is an integrated viewer that uses state of the art phasing and tag SNP selection algorithms. It streamlines the application of GERBIL and STAMPA, which were available until now only as batch executables, and allows using them together with the strong visualizations of Haploview for assessment of the results. Both running the algorithms and visualizing the results are done within the graphical user interface, unlike, e.g., the HapScope software [6], which only enables the latter. This makes the algorithms accessible to the broad community of researchers in genetics.

## Availability and requirements

• **Project name**: GEVALT

• **Project home page**: 

• **Operating systems**: Windows and Linux.

• **Programming language**: Java and C++

• **Other requirements**: Java 1.3 or higher.

• **License**: free non-commercial research use license.

• **Any restrictions to use by non-academics**: license needed for commercial use.

## Authors' contributions

OD and GK contributed to the design of GEVALT. OD implemented GEVALT and GK implemented the analysis algorithms. RS supervised the project. All authors participated in the drafting and revising of the manuscript, and read and approved the final manuscript.
